# A Preclinical Immunogenicity Study of the Recombinant Human Papillomavirus Nine-Valent Virus-like Particle Vaccine

**DOI:** 10.3390/vaccines12121356

**Published:** 2024-11-30

**Authors:** Dan Xu, Jia-Dai Li, Jiao An, Xin-Xing Ma, Xiao-Liang Wang, Zheng Zhou, Hai-Ping Liu, Mei-Jun Diao, Yuan-Xiang Jiang, Ling-Yun Zhou, Xin Tong, Chen-Liang Zhou

**Affiliations:** 1 Shanghai Zerun Biotech Co., Ltd., Building 9, 1690 Zhangheng Road, Pudong, Shanghai 201203, China; xudan@walvax.com (D.X.); lijiadai@walvax.com (J.-D.L.); anjiao@walvax.com (J.A.); maxinxing@walvax.com (X.-X.M.); wangxiaoliang@walvax.com (X.-L.W.); zhouzheng@walvax.com (Z.Z.); liuhaiping@walvax.com (H.-P.L.); diaomeijun@walvax.com (M.-J.D.); jiangyuanxiang@walvax.com (Y.-X.J.); zhoulingyun@walvax.com (L.-Y.Z.); 2 Yunnan Walvax Biotech Co., Ltd., No. 395 Kexin Road, Wuhua District, Kunming 650021, China

**Keywords:** human papillomavirus (HPV), virus-like particles (VLPs), vaccines, immunogenicity, protective efficacy

## Abstract

Background: Cervical cancer is associated with persistent infection of high-risk human papillomaviruses (HPVs). Prophylactic HPV vaccines have been recommended and have significant efficacy in preventing cervical cancer. Multivalent HPV vaccines have a better preventative effect on HPV-related diseases. However, there is currently only one nine-valent HPV vaccine on the market: Gardasil^®^ 9. The development of new HPV vaccines is still urgent in order to achieve the goal of eliminating cervical cancer as proposed by the WHO. Methods: In this study, we developed a nine-valent recombinant HPV virus-like particle (VLP) vaccine (HPV-9 vaccine) containing HPV type 6, 11, 16, 18, 31, 33, 45, 52, and 58 antigens, with an adjuvant of aluminum phosphate (AlPO_4_). The type-specific L1 proteins were recombinantly expressed using *Pichia pastoris*, followed by self-assembly into VLPs. Immunogenicity studies of the HPV-9 vaccine were performed using rodents (mice and rats) and non-human primates (macaques) as animal models. Results: Immunogenicity studies showed that the HPV-9 vaccine is able to elicit a robust and long-lasting neutralizing antibody response in rodents (mice and rats) and non-human primates (cynomolgus macaque) models. The HPV-9 vaccine shows immunogenicity comparable to that of Walrinvax^®^ and Gardasil^®^ 9. Conclusions: In summary, this study provides a comprehensive investigation of the immunogenicity of the HPV-9 vaccine, including its immune persistence. These findings, derived from using models of diverse animal species, contribute valuable insights into the potential efficacy of the vaccine candidate in clinical settings.

## 1. Introduction

Human papillomavirus (HPV), a non-enveloped double-stranded DNA virus measuring approximately 44~55 nm in diameter, represents the most prevalent sexually transmitted infection. It has been identified that 15 types of HPV are associated with the development of various cancers, including cervical, anal, oropharyngeal, penile, vulvar, and vaginal cancers [1,2]. Notably, persistent infection with high-risk HPVs is responsible for nearly 99% of cervical cancer cases. Despite the global incidence of cervical cancer ranking third among women, with an estimated 661,021 new cases and 348,189 deaths in 2022, it is important to acknowledge that the majority of HPV infections resolve spontaneously without symptoms within approximately 6 to 24 months [3]. However, a small subset of these infections progresses to cervical carcinogenesis, following a protracted evolutionary course encompassing HPV acquisition, persistent infection, the development of precancerous lesions, and, ultimately, invasive cancer [4]. Recognizing the health burden, the World Health Organization (WHO) has endorsed HPV vaccination as a primary preventive measure. In 2018, WHO called for the elimination of cervical cancer globally and, in 2019, proposed a strategy for 2020–2030 [5].

Presently, six prophylactic HPV vaccines are available on a global scale. These include three bivalent vaccines (Cervarix^®^ by GlaxoSmithKline Biologicals, London, UK, Cecolin^®^ by Xiamen Innovax Biotech, Xiamen, China, and Walrinvax^®^ by Yuxi Zerun Biotech, Yuxi, China) targeting HPV16 and 18, two quadrivalent vaccines (Gardasil^®^ by Merck & Co., Rahway, NJ, USA and Cervavac^®^ by Serum Institute of India, Pune, India), covering HPV6, 11, 16, and 18, and Gardasil^®^ 9(Merck & Co., Rahway, NJ, USA), a nine-valent vaccine approved by the FDA in 2014, which protects against HPV6, 11, 16, 18, 31, 33, 45, 52, and 58. Notably, HPV6 and 11 cause approximately 90% of genital warts cases. All of these vaccines are formulated utilizing the noninfectious recombinant L1 capsid protein, which is the major protein constituent of the HPV viral capsid. The recombinant L1 protein is produced through various expression systems, and upon assembly, it forms virus-like particles (VLPs) that closely resemble natural HPV virions. This assembly process confers the VLP antigen with exceptional immunogenicity [6,7,8,9,10,11]. Although Gardasil^®^ 9 was approved in 2014, as of 2023, only 27% of females worldwide have received a first dose of the HPV vaccine [12]. To meet WHO’s cervical cancer elimination goal, the nine-valent vaccine production capacity must be increased, especially for low- and middle-income countries, including China.

We have successfully developed a bivalent vaccine named Walrinvax^®^, targeting HPV16 and HPV18. Walrinvax^®^ has demonstrated very good safety and immunogenicity in clinical trials [13,14,15]. Walrinvax^®^ was approved by the Chinese National Medical Products Administration (NMPA) in 2022 and received WHO prequalification in 2024 [16]. Extending this research, recombinant L1 proteins for HPV types 6, 11, 31, 33, 45, 52, and 58 were produced using the *Pichia pastoris* expression system. These proteins underwent self-assembly into VLPs and were adsorbed onto aluminum phosphate (AlPO_4_) adjuvant to develop a nine-valent HPV vaccine. This vaccine, comprising HPV16, 18, 6, 11, 31, 33, 45, 52, and 58 demonstrated comparable immunogenicity to Gardasil^®^ 9. Currently, this vaccine candidate is undergoing phase III clinical trials in China (NCT05580341).

## 2. Materials and Methods

### 2.1. Cells

293FT cells (GIBCO, Grand Island, NY, USA) were maintained in a controlled laboratory environment. The cells were cultured in Dulbecco’s modified Eagle’s medium (DMEM) (GIBCO, USA), supplemented with 10% fetal bovine serum (FBS) (GIBCO, USA) and 1% penicillin-streptomycin solution (Shanghai Yuanpei Biotech, Shanghai, China). The culture conditions included an incubation temperature of 37 °C, a 5% concentration of CO_2_, and a high-humidity environment.

### 2.2. Construction of Expression Plasmids and Screening of Expression Strain

The amino acid sequences of L1 proteins were retrieved from Genbank with the following accession numbers: NP_040304.1 for HPV 6L1, AAA46935.1 for HPV 11L1, AAC09292.1 for HPV 16L1, AAP20601 for HPV 18L1, AEI60965.1 for HPV 31L1, ACL12333.1 for HPV 33L1, ABP99831.1 for HPV 45L1, AIF71444.1 for HPV 52L1, and BAA31851.1 for HPV 58L1. Optimized genes encoding these L1 proteins were synthesized by Generay Biotech (Shanghai, China) and then cloned into the yeast expression vectors pPIC Z*α*A or pPIC Z*α*B using the restriction enzymes BstBI (NEB, Ipswich, MA, USA) and KpnI (NEB, Ipswich, MA, USA). After the successful construction of the expression plasmids, the L1 expression plasmids were linearized using the restriction enzyme SacI (NEB, Ipswich, MA, USA) prior to electro-transformation into *Pichia pastoris* strains. *Pichia pastoris* strains exhibiting high expression levels were selected using selective antibiotics, specifically Zeocin (Gibco, Carlsbad, CA, USA).

### 2.3. Protein Expression, Purification and Structural Analysis

Following the screening of strains, the expression of L1 proteins was induced using methanol (Sinopharm, Shanghai, China). To confirm the expression of L1 proteins, Western blot analysis was performed using anti-L1 mouse antibodies, which were prepared in-house, along with HRP-conjugated goat anti-mouse IgG(H+L) (BioRad, Hercules, CA, USA) as the secondary antibody. Subsequently, the purification of L1 proteins was carried out using POROS 50HS cation exchange resin (ThermoFisher, Carlsbad, CA, USA) and CHT-II resin (BioRad, Hercules, CA, USA). The purified L1 proteins were analyzed using SDS-PAGE with 12% polyacrylamide gels, and the protein molecular weight was determined using an unstained protein MW marker (ThermoFisher, MA, USA). Since L1 proteins possess the ability to self-assemble into VLPs, structural characterization was conducted using various methods. Analytical ultracentrifugation (AUC) was performed using equipment from Beckman Coulter (Brea, CA, USA) to study the sedimentation properties of the VLPs. A differential scanning calorimeter (Malvern, WR14 1XZ, UK) was used to investigate the thermal stability of the VLPs. A dynamic light scattering instrument (Malvern, WR14 1XZ, UK) was utilized to determine the size distribution of the VLPs. Additionally, negative staining transmission electron microscopy (TEM) was conducted using a JEM-JEOL microscope (JEOL, Tokyo, Japan) to visualize the morphology of the VLPs.

### 2.4. Formulation of the HPV-9 Candidate

We prepared the aluminum phosphate adjuvant (AlPO_4_) in-house and utilized it to adsorb HPV type 6, 11, 16, 18, 31, 33, 45, 52, and 58 L1 VLPs individually. For our immunogenicity study of the monovalent HPV VLPs, the AlPO_4_-absorbed VLPs were diluted to concentrations of 0.4 µg/mL, 0.04 µg/mL, and 0.004 µg/mL using an AlPO_4_ adjuvant with a concentration of 1 mg/mL. The final formulation of the HPV-9 candidate consisted of 30 μg HPV6 L1 VLPs, 40 μg HPV11 L1 VLPs, 60 μg HPV16 L1 VLPs, 40 μg HPV18 L1 VLPs, 20 μg HPV31 L1 VLPs, 20 μg HPV33 L1 VLPs, 20 μg HPV45 L1 VLPs, 20 μg HPV52 L1 VLPs, and 20 μg HPV58 L1 VLPs, along with 500 μg of AlPO_4_ adjuvant per 0.5 mL of vaccine formulation. The commercial HPV vaccine Gardasil^®^ 9 was from MSD for comparison and reference purposes.

### 2.5. Enzyme-Linked Immunosorbent Assay (ELISA)

Binding antibodies were measured using the enzyme-linked immunosorbent assay (ELISA) methodology [9]. Briefly, 96-well microplates were coated with 100 ng of individual HPV VLPs per well and incubated overnight at 4 °C. Subsequently, the plates were washed one time with PBS containing 0.05% Tween 20 (PBST), followed by blocking with PBST containing 5% skim milk for two hours at 37 °C.

Heat-inactivated serum samples were serially diluted with PBST containing 2% skim milk, the diluted serums were subsequently added into plates with a volume of 100 μL per well and incubated for one hour at 37 °C. Horseradish peroxidase (HRP)-conjugated goat anti-mouse IgG (BioRad, Hercules, CA, USA) was diluted with PBST containing 2% skim milk at a ratio of 1:10,000 and added to plates with a volume of 100 μL per well, followed by incubation for one hour at 37 °C.

After incubation, the plates were washed six times with PBST to remove any unbound materials. A total of 100 μL per well of the TMB substrate system solution (SeraCare, Milford, MA, USA) was added, and the plates were incubated for ten minutes in the dark at 37 °C. The reaction was stopped by adding 50 μL per well of 2 M sulfuric acid. The absorbance of the samples was measured at 450 nm and 620 nm using a microplate reader (SpectraMax M5, MD, San Jose, CA, USA). The endpoint titer was determined as the reciprocal of the highest serum dilution that produced an optical density (OD) reading at 450 nm–620 nm, which was at least 2.1-fold higher than the negative control.

### 2.6. The Preparation of the Pseudoviruses of the Nine HPV Types

The preparation of the pseudoviruses and neutralizing assay was conducted as previously described [17]. Plasmids encoding L1 and L2 capsid proteins of HPV types 6, 11, 16, 18, 31, 33, 45, 52, or 58 were separately synthesized and cloned into the pcDNA3.1(-) vector by reputable companies, including Generay Biotech (Shanghai, China), Sangon (Shanghai, China), and Genscript (Nanjing, China). The zsGreen reporter gene was also synthesized and cloned into the pcDNA3.1(-) vector. Report gene plasmids including pRFP and pCFP were kindly gifted by the National Institutes for Food and Drug Control (NIFDC). The plasmids encoding each type of capsid protein, along with reporter plasmids pZsGreen, pRFP, or pCFP, were co-transfected into 293FT cells using Lipofectamine2000. The transfected cells were then subjected to incubation at 37 °C with 5% CO_2_ for 72 h. Subsequently, the transfected cells were treated with 0.25% Trypsin-EDTA (Gibco, CA, USA), and the resulting cell pellet was suspended in PBS. The suspension was then treated with Brij58 (Sigma, Saint Louis, MO, USA) and Benzonase Nuclease (Novagen, Burlington, MA, USA) overnight at 37 °C to allow for the maturation of the pseudoviruses. The pseudoviruses were separated from the cell lysate through centrifugation at 1000 rpm for 5 min and stored at −80 °C for use. To determine the 50% tissue culture infectious dose (TCID_50_) of each type of pseudovirus, 10-fold serial dilutions of pseudovirus for each HPV type were added into a 96-well cell culture plate containing 1.5 × 10^4^ 293FT cells per well in a total volume of 100 µL, with 8 replicates for each dilution. The plate was incubated at 37 °C with 5% CO_2_ for 72 h. After the incubation period, the fluorescence signal was quantified via the use of either a fluorescence microscope (Olympus, Tokyo, Japan) or a fluorescence ELISPOT analyzer (CTL, Cleveland, OH, USA). The TCID_50_ was calculated using the Reed–Muench method.

### 2.7. Pseudovirus-Based Neutralizing Assay

Neutralizing antibody titers induced by the HPV-9 candidate were assessed via pseudovirus-based neutralizing assays conducted using 293FT cells. The 293FT cells were seeded in a 96-well cell culture plate at a density of 1.5 × 10^4^ cells per well in a total volume of 100 µL. Mouse, rat, or monkey sera were heat-inactivated at 56 °C for 30 min and subjected to two-fold serial dilutions. For our single-color pseudovirus-based neutralization assay, type-specific pseudovirus was individually diluted to a concentration of lg (TCID_50_/0.1 mL) = 3.2 ± 0.5. For our triple-color pseudovirus-based neutralization assay, pseudovirus was diluted with a certain ratio according to the TCID_50_; then, the corresponding pseudovirus was mixed in equal volume based on the combination of pRFP and pCFP. Equal volumes of the diluted pseudovirus (70 µL) and the serially diluted sera were mixed and incubated at room temperature for 60 min. As a negative control, equal volumes of pseudovirus and culture medium were mixed (70 µL). Subsequently, 100 µL of the pseudovirus–serum mixture or the negative control was added to the 96-well cell culture plate, with two replicates for each dilution. The plate was then incubated at 37 °C with 5% CO_2_ for 72 h. Following the incubation period, the fluorescence signal was analyzed using either fluorescence microscopy or a fluorescence ELISPOT analyzer to determine the percentage of infection inhibition. The endpoint titers were calculated as the highest serum dilution that resulted in a percentage of infection inhibition greater than 50% compared to the negative control. All samples were analyzed individually, and the values presented here represent the geometric mean of all replicates.

### 2.8. Animal Studies

Multiple animal models have been used to evaluate the immunogenicity and immune persistence of HPV-9, including rodent models and non-human primate models. Studies in BALB/c mice and SD rats were carried out in compliance with the guidelines and protocols approved by the Institutional Animal Care and Use Committee (IACUC) of Shanghai Bikai-Keyi Biotech (Shanghai, China) (IACUC: O01002-03, O01002-07, O01002-10). Our macaque-based immunogenicity study was approved by the Animal Research Ethics Board of United-Power Pharma Tech Co., Ltd. (Nanning, Guangxi, China) (IACUC: GD20181202).

For our immunogenicity study of the nine monovalent HPV vaccines, female BALB/c mice aged six to eight weeks were obtained from Shanghai Bikai-Keyi Biotech (Shanghai, China) and housed under specific-pathogen-free (SPF) conditions. The mice were randomly divided into groups, with there being 10 mice in each group, before vaccination. The mice in each group were intraperitoneally injected with 0.5 mL of monovalent vaccines containing 0.002 µg, 0.02 µg, or 0.2 µg of monovalent VLPs at weeks 0 and 2, and mice injected with 0.5 mL of the AlPO_4_ adjuvant twice at a two-week interval were serving as the placebo control. The mice in each group were bled and then sacrificed at week 4. Serum samples were collected and heat-inactivated at 56 °C for 30 min to evaluate antibody responses via ELISAs and neutralization assays.

For the mice-based immunogenicity study, female BALB/c mice aged six to eight weeks obtained from Shanghai Bikai-Keyi Biotech (Shanghai, China) were housed under specific-pathogen-free (SPF) conditions and randomly divided into groups, with there being 10 animals in each group, followed by intraperitoneal injection of 0.5 mL of 100-fold-diluted HPV-9 vaccine, Gardasil^®^ 9, or Walrinvax^®^ at weeks 0, 2, and 6. The mice injected three times with 0.5 mL of the AlPO_4_ adjuvant served as the placebo control. Serum samples were collected at weeks 2, 4, 8, 12, 20, 24, and 28. The mice were sacrificed after the final bleeding at week 28. Serum samples from each timepoint were heat-inactivated at 56 °C for 30 min to perform neutralization assays to evaluate the immune response induced by the HPV-9 vaccine.

For the immunogenicity study in Sprague Dawley (SD) rats, female SD rats with a body weight of 120–150 g were obtained from Shanghai Bikai-Keyi Biotech (Shanghai, China) and housed under specific pathogen-free (SPF) conditions. The rats were randomly divided into groups, with there being 5 animals in each group. The rats were intramuscularly injected with 0.5 mL of 10-, 100-, or 1000-fold-diluted vaccines including the HPV-9 vaccine, Gardasil^®^ 9, or Walrinvax^®^ at weeks 0, 2, and 6, while rats intramuscularly injected with the same volume of the AlPO_4_ adjuvant were serving as the placebo control. Serum samples were collected at weeks 2, 4, 8, 12, 20, 24, and 28. The rats were sacrificed after the final bleeding at week 28. Serum samples from each timepoint were heat-inactivated at 56 °C for 30 min to measure the neutralizing antibody titers against the nine HPV types.

Six female and six male macaques aged 2 to 3 years with bodyweight values of 2 to 3 kg were purchased from Guangxi Guidong Primate Development Experiment Co., Ltd. (Wuzhou, Guangxi, China), and the macaque-based immunogenicity study was conducted by United-Power Pharm Tech (UP Pharm, Beijing, China). All these macaques were negative for TB, B virus, SRV, SIV, and STLV-1, and the neutralizing antibody titers against the 9 types of HPV were less than 50. The macaques were randomly divided into two groups, with 3 females and 3 males in each group. The macaques were intramuscularly injected with 0.5 mL of the HPV-9 vaccine or Gardasil^®^ 9 at weeks 0, 8, and 24. The macaques were bled at weeks 0 (before the first injection), 4, 12, and 26. Serum samples collected at weeks 4, 12, and 26 were heat-inactivated at 56 °C for 30 min to evaluate the immune responses induced by the HPV-9 vaccine in non-human primate animals. The macaques in each group were euthanized by bleeding from the inguinal artery as they were deeply anesthetized through intravenous injection of Zoletil^®^ 50 and Xylazine (1:1) at a dosage of 0.2 mL/kg, and this procedure was strictly conducted according to the “Laboratory Animal-Guidelines for Euthanasia” and approved by the Chinese Standardization Administration.

### 2.9. Statistical Analyses

All statistical analyses were conducted using GraphPad Prism 10.4. Antibody titers were expressed as the geometric mean with a 95% confidence interval (CI). Differences between groups were evaluated using a two-way ANOVA (Version 10.4.0 (621)), followed by Tukey’s multiple comparisons test. A *p*-value less than 0.05 was considered statistically significant (*p* > 0.05 (ns), ≤0.05 (*), ≤0.01 (**), ≤0.001 (***), and <0.0001 (****)).

## 3. Results

### 3.1. Recombinant Expression of HPV L1s and Structural Characterization of VLPs

**Figure 1:**
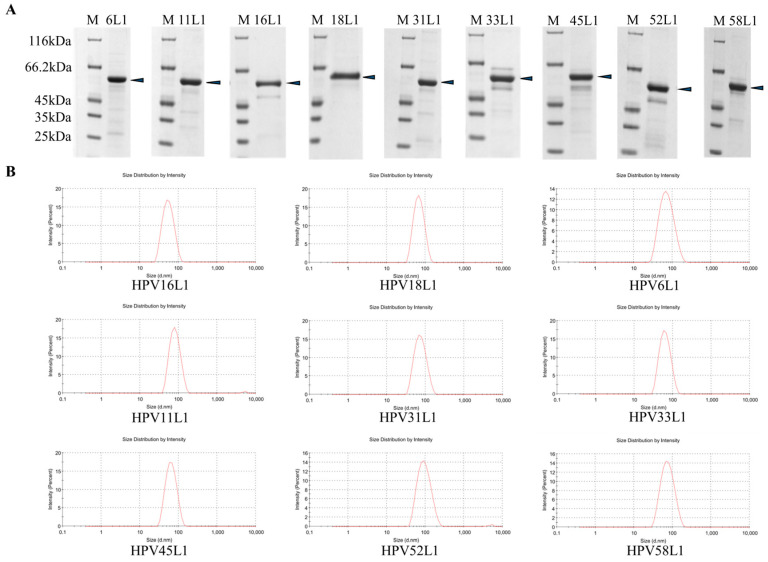

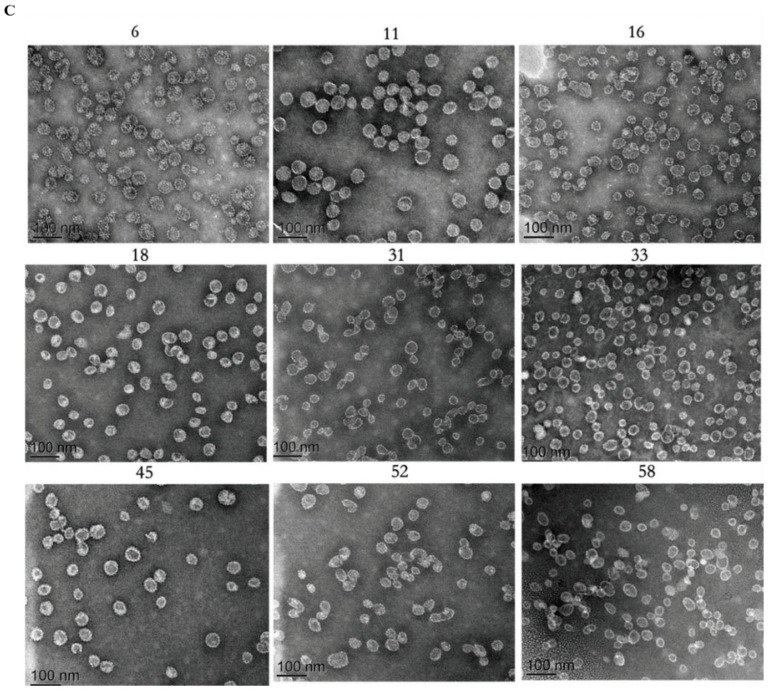
Characterization of recombinant expressed HPV L1 proteins. (**A**) Recombinant expressed HPV L1 proteins of each type were purified and subjected to reduced SDS-PAGE, arrowheads indicated the major band of HPV L1s. (**B**) Purified HPV L1 proteins were self-assembled into VLPs, and the nano-particle size was detected through using the Zetasizer Nano instrument. (**C**) Self-assembled VLPs of all nine types were characterized through transmission electron microscopy (TEM).

HPV L1 proteins were expressed in *Pichia pastoris*, and their expression level was assessed via Western blot analysis. The purified HPV L1 proteins for each HPV type were analyzed using SDS-PAGE, which revealed distinct bands with an apparent molecular weight of approximately 55 kDa, as shown in Figure 1A. Furthermore, comprehensive structural characterization of the self-assembled VLPs was performed. This included the determination of nano-particle size, as demonstrated in Figure 1B, and visualization using transmission electron microscopy (TEM), as shown in Figure 1C. These data collectively demonstrated that the recombinant expression of HPV L1 proteins in the *Pichia pastoris* expression system yielded prominent bands at the expected molecular weight of ~55 kDa. Importantly, the recombinant L1 proteins exhibited the ability to self-assemble into VLPs with a nano-size range of 50~80 nm.

Subsequently, these self-assembled VLPs were utilized as components for the HPV-9 vaccine, with aluminum phosphate (AlPO_4_) serving as the adjuvant. The nine-valent VLPs were adsorbed to the adjuvant individually, followed by the formulation of the HPV-9 vaccine through mixing these adsorbed monovalent VLPs in a specific ratio.

### 3.2. Immunogenicity Study of Monovalent HPV Vaccines in Mice

**Figure 2:**
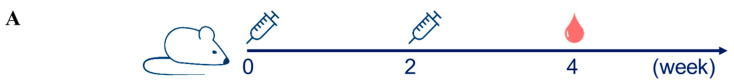

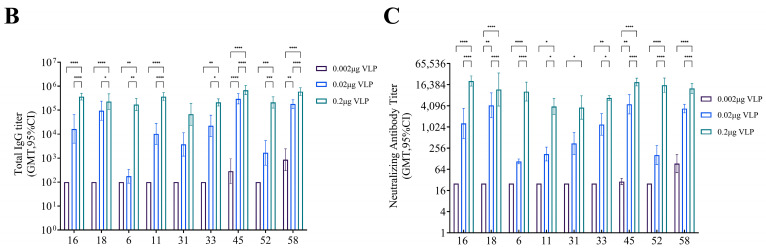
Immunogenicity study of the nine monovalent AlPO_4_-adsorbed HPV VLPs. (**A**) Scheme of mice-based immunization routine. Briefly, female BALB/c mice were randomly divided into groups (with 10 mice/group), followed by intraperitoneal injection of 0.002 μg, 0.02 μg, or 0.2 μg monovalent AlPO_4_-adsorbed VLPs twice at a 2-week interval, with injection of the adjuvant AlPO_4_ alone as the placebo. Serum samples were collected for detection of binding antibody titers and neutralizing antibody titers. (**B**) Binding antibody titers were detected through ELISAs using HPV VLPs of each type as antigens, serum from immunized mice as the 1st antibody with series dilutions, and diluted HRP-conjugated Goat anti-Mouse (H+L) IgG as the 2nd antibody. *p*-value less than 0.05 was considered statistically significant (*p* ≤ 0.05 (*), ≤0.01 (**), ≤0.001 (***), and <0.0001 (****)). (**C**) Detection of neutralizing antibody titers was conducted based on the HPV pseudovirus of each type we prepared. *p*-value less than 0.05 was considered statistically significant (* *p* ≤ 0.05 (*), ≤0.01 (**), ≤0.001 (***), and <0.0001 (****)).

In order to evaluate the immunogenicity of the formulated monovalent AlPO_4_-adsorbed VLPs targeting HPV6, 11, 16, 18, 31, 33, 45, 52, and 58, an immunogenicity study was conducted in BALB/c mice. Briefly, 0.002 μg, 0.02 μg, or 0.2 μg of these monovalent AlPO_4_-adsorbed VLPs was intraperitoneally injected into the mice twice at a 2-week interval, with injection of the adjuvant alone as the placebo control. Two weeks after the second injection, serum samples were collected to assess the levels of antigen-specific binding antibodies and neutralizing antibodies, thereby evaluating the immunogenicity of the recombinant HPV VLPs (Figure 2A). The results shown in Figure 2B,C reveal that the nine AlPO_4_-adsorbed HPV VLPs elicited high titers of both binding and neutralizing antibodies compared to the placebo control. Furthermore, a significant dose-response relationship was observed across different immune doses, indicating a dose-dependent effect. These findings demonstrate that the recombinant HPV VLPs possess excellent immunogenicity, thereby supporting their potential as key components in the development of the HPV-9 vaccine.

### 3.3. Immunogenicity and Immune Persistence Study of HPV-9 in Mice

**Figure 3:**
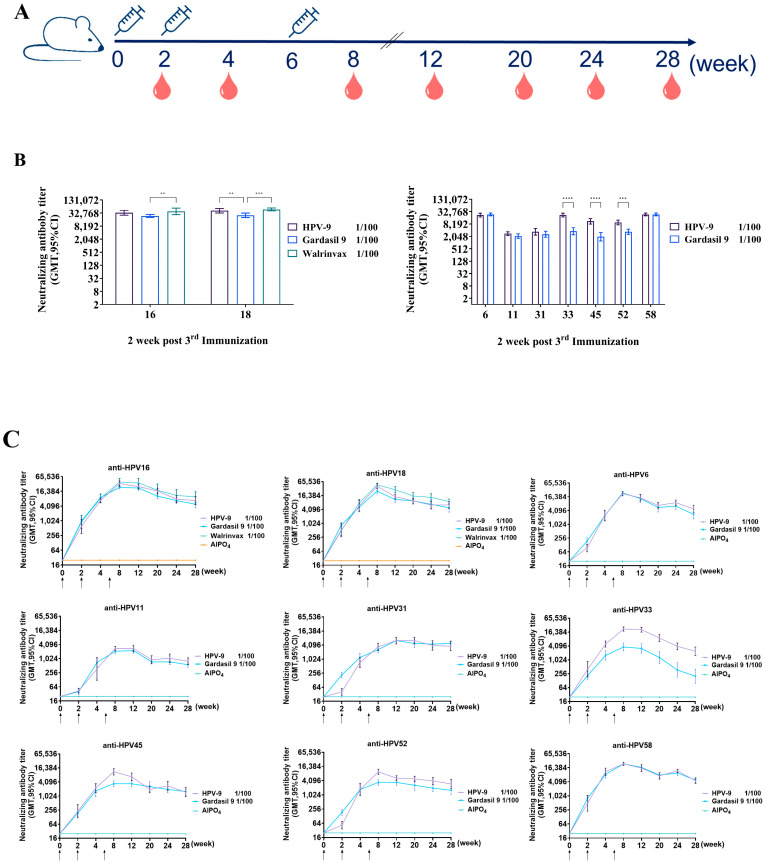
The immunogenicity and immune persistence of the HPV-9 vaccine in BALB/c mice. (**A**) As the immunization routine scheme shows, female BALB/c mice were randomly divided into groups, with 10 mice per group. The mice were intraperitoneally injected with a 1/100 clinical dose of Gardasil^®^ 9 and HPV-9 vaccine or Walrinvax^®^ or the adjuvant as the placebo at weeks 0, 2, and 6; neutralizing assays were conducted at weeks 2, 4, 8, 12, 20, 24 and 28. (**B**) Two weeks after the 3rd vaccination, the neutralizing antibody titers tested through neutralizing experiments demonstrated that the HPV-9 vaccine’s effectiveness is comparable to that of Gardasil^®^ 9 and Walrinvax^®^ (*p* ≤ 0.01 (**), ≤0.001 (***), and <0.0001 (****)). (**C**) To evaluate the long-term protection induced by the HPV-9 vaccine in comparison with Gardasil^®^ 9 or Walrinvax^®^, neutralizing assays were conducted to test the antibody-specific neutralizing antibodies in serum from vaccinated mice. The arrowheads represent the immunization timepoints.

Having confirmed the immunogenicity of the recombinant HPV VLPs, the HPV-9 vaccine was formulated with the nine AlPO_4_-adsorbed VLPs, containing 270 μg of HPV L1 proteins per 0.5 mL. To assess the immunogenicity and immune persistence, the study was conducted in BALB/c mice using intraperitoneal injection of 1/100 clinical dose of the HPV-9 vaccine, Gardasil^®^ 9, or Walrinvax^®^ (Figure 3A). Neutralization assays revealed that three doses of HPV-9 induced maximum neutralizing antibody titers against HPV types 6, 11, 16, 18, 31, 33, 45, 52, and 58 at week 8, with values of 22,286; 2786; 33,779; 41,587; 3430; 22,286; 11,143; 9701; and 23,886, respectively. In the Gardasil^®^ 9 group, the corresponding maximum titers were 23,886; 2111; 23,886; 25,600; 2599; 3676; 1970; 3430; and 23,886. Notably, higher titers against HPV 16 and 18 were induced by Walrinvax^®^, reaching 38,802 and 47,771, respectively. These findings demonstrate that the HPV-9 vaccine induces comparable immunogenicity to both Gardasil^®^ 9 and Walrinvax^®^ (Figure 3B).

Neutralizing antibody titers followed a consistent trend across vaccine groups, increasing significance after the second vaccination, peaking at week 8 (2 weeks after the third vaccination), and gradually declining while maintaining high levels until week 28. Neutralization antibodies against HPV16, induced via a 1/100 clinical dose of the HPV-9 vaccine, Walrinvax^®^, or Gardasil^®^ 9, found values of 6859, 9701 and 4850, with *p*-values of 0.8529 (HPV-9 vs. Gardasil^®^ 9) and 0.1598 (Walrinvax^®^ vs. Gardasil^®^). Neutralization antibodies against HPV18, elicited via a 1/100 clinical dose of the HPV-9 vaccine, Walrinvax^®^, or Gardasil^®^ 9, found values of 6400, 9051 and 4850, with *p*-values of 0.9337 (HPV-9 vs. Gardasil^®^ 9) and 0.3522 (Walrinvax^®^ vs. Gardasil^®^ 9). For HPV6 and HPV11, the HPV-9 vaccine-induced titers of 4850 and 800, compared to 2786 and 566 for Gardasil^®^ 9 (*p* = 0.2896 and 0.4554, respectively). Neutralizing antibodies against HPV31 and HPV33 induced by the HPV-9 vaccine were 3676 and 2425, compared to 4850 and 200 for Gardasil^®^ 9, with a significant difference for HPV33 (*p* = 0.0390). For HPV45 and HPV52, the HPV-9 vaccine generated titers of 1493 and 2986, compared to those of 1600 and 1600 for Gardasil^®^ 9 (*p* = 0.9943 and 0.0107, respectively). For HPV58, both vaccines showed similar titers (4525 and 4850, *p* = 0.9706) (Figure 3C).

### 3.4. Studying the Immunogenicity and Immune Persistence of the HPV-9 Vaccine in SD Rats

**Figure 4:**
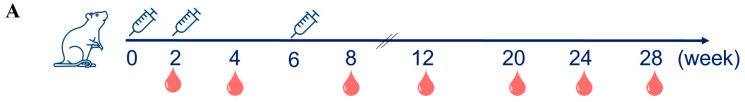

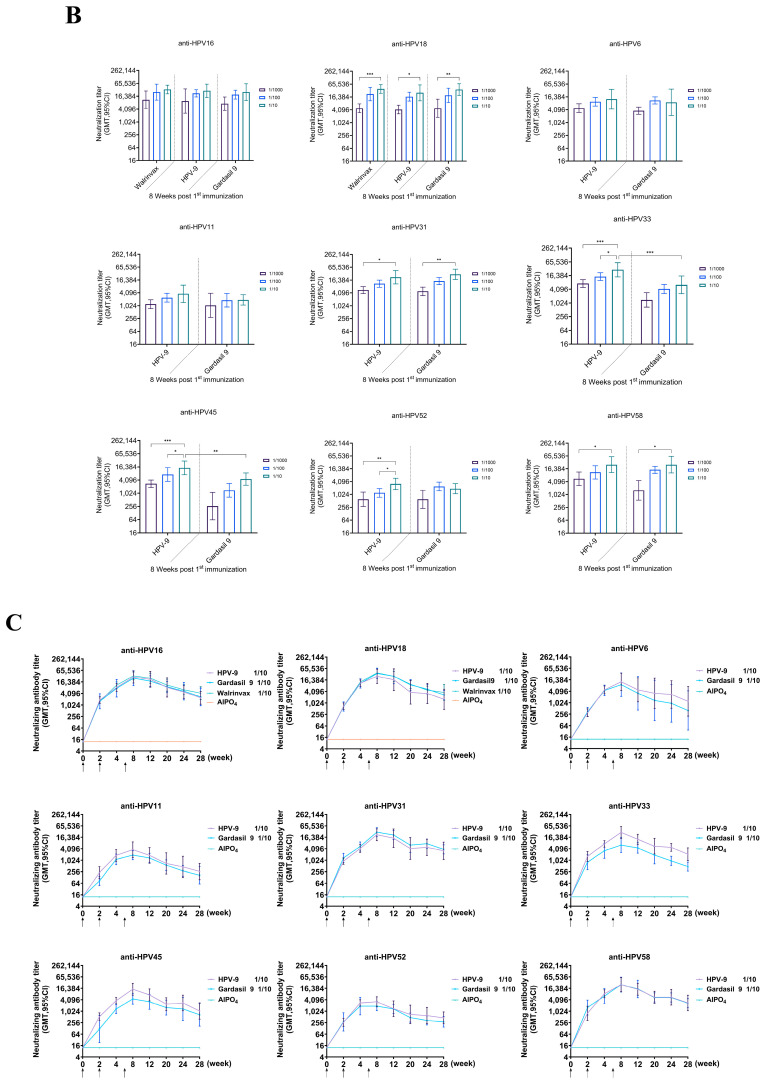
The immunogenicity and immune persistence of the HPV-9 vaccine in SD rats. (**A**) As the immunization routine scheme shows, female SD rats were randomly divided into groups (5 rats per group) and then intramuscularly injected with vaccines, namely the HPV-9 vaccine, Walrinvax^®^, and Gardasil^®^ 9, at 1/10, 1/1000, or 1/10,000 of the clinical dose, respectively, at weeks 0, 2, and 6. Rats injected with the AlPO_4_ adjuvant alone were used as the placebo control. Neutralizing assays of serum samples were conducted at weeks 2, 4, 8, 12, 20, 24, and 28. (**B**) Two weeks after the 3rd vaccination, the neutralizing antibody levels tested through neutralizing experiments showed that all vaccines induced dose-dependent immunogenicity, which was comparable among the vaccines (*p* ≤ 0.05 (*), ≤0.01 (**), and ≤0.001 (***)). (**C**) To evaluate the long-term protective effect induced by the HPV-9 vaccine compared to Gardasil^®^ 9 or Walrinvax^®^, neutralizing assays were conducted to test the antibody-specific neutralizing antibodies in serum from vaccinated rats. Durable immune protection was induced with 1/10 of the clinical dose. The arrowheads represent the vaccination timepoints.

Furthermore, we conducted an immunogenicity and immune persistence study using an SD rat model. The rats received intramuscular injections of 1/1000, 1/100, or 1/10 clinical doses of the HPV-9 vaccine, Gardasil^®^ 9, or Walrinvax^®^ (Figure 4A). The results demonstrated a notable dose-dependent effect in all vaccine groups. Vaccination with a 1/1000 clinical dose of the HPV-9 vaccine three times, resulted in neutralizing antibodies against HPV types 6, 11, 16, 18, 31, 33, 45, 52, and 58 at levels of 4850; 1213; 9701; 4222; 5572; 7352; 2786; 606; and 5572, respectively. For Gardasil^®^ 9, the titers were 3676; 1056; 7352; 4850; 4850; 1393; 264, 606; and 1600 (Figure 4B). Significantly higher levels of neutralizing antibodies against HPV 33 and HPV 45 were observed in the HPV-9 group compared to the Gardasil^®^ 9 group. At higher doses, this difference was more pronounced. For 1/10 clinical dose, the HPV-9 vaccine-induced titers of 29,407 and 14,703 against HPV33 and HPV45, compared to 6400 and 4525 for Gardasil^®^ 9, with *p*-values of 0.0006 and 0.0032, respectively. These findings highlight the dose-dependent immune response and the enhanced immunogenicity of the HPV-9 vaccine, particularly highlighting its ability to generate higher neutralizing antibody levels against specific HPV types compared to the control vaccine.

To assess the immune persistence of the HPV-9 vaccine, we measured the titers of neutralizing antibodies at weeks 2, 4, 8, 12, 20, 24, and 28. Notably, the maximum titers against HPV types 6, 11, 16, 18, 31, 33, 45, 52, and 58 were observed at week 8 when administering 1/10 of the clinical dose of the HPV-9 vaccine. Specifically, the neutralization antibodies reached values of 12,800; 3676; 29,407; 25,600; 22,286; 29,407; 14,703; 3200; and 25,600; respectively. Importantly, these neutralizing antibody levels remained consistently high up to 28 weeks after the final vaccination (Figure 4C). Both the HPV-9 vaccine and Gardasil^®^ 9 exhibited a dose-effect relationship, with increasing neutralizing antibody levels corresponding to the immune dose administered. In terms of immune persistence, the HPV-9 vaccine maintained comparable neutralizing antibody levels to those of Gardasil^®^ 9 for HPV types 6, 11, 16, 18, 31, 52, and 58 (*p* > 0.05). However, the levels of neutralizing antibodies against HPV 33 and HPV 45 induced by the HPV-9 vaccine were significantly higher than those induced by Gardasil^®^ 9 (*p* < 0.05), thrice vaccination of the HPV-9 vaccination or Gardasil^®^ 9 with 1/10 of the clinical dose induced anti-HPV 33 neutralizing antibody titers of 29,407 and 6400 with a *p*-value of 0.0006, and anti-HPV45 neutralizing antibody titers of 14,703 and 4525 with a *p*-value of 0.0032. These results were consistent with our findings in the BALB/c mouse model. Taken together, the HPV-9 vaccine exhibited high immunogenicity and immune persistence, particularly for HPV33 and HPV45, making it comparable to Gardasil^®^ 9.

### 3.5. Comparing the Immunogenicity of the HPV 9 Vaccine and Gardasil^®^ 9 in Macaques

**Figure 5:**
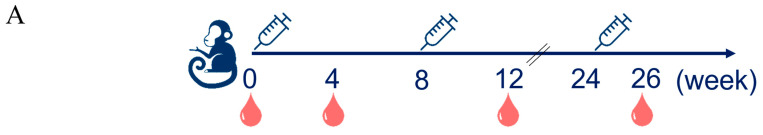

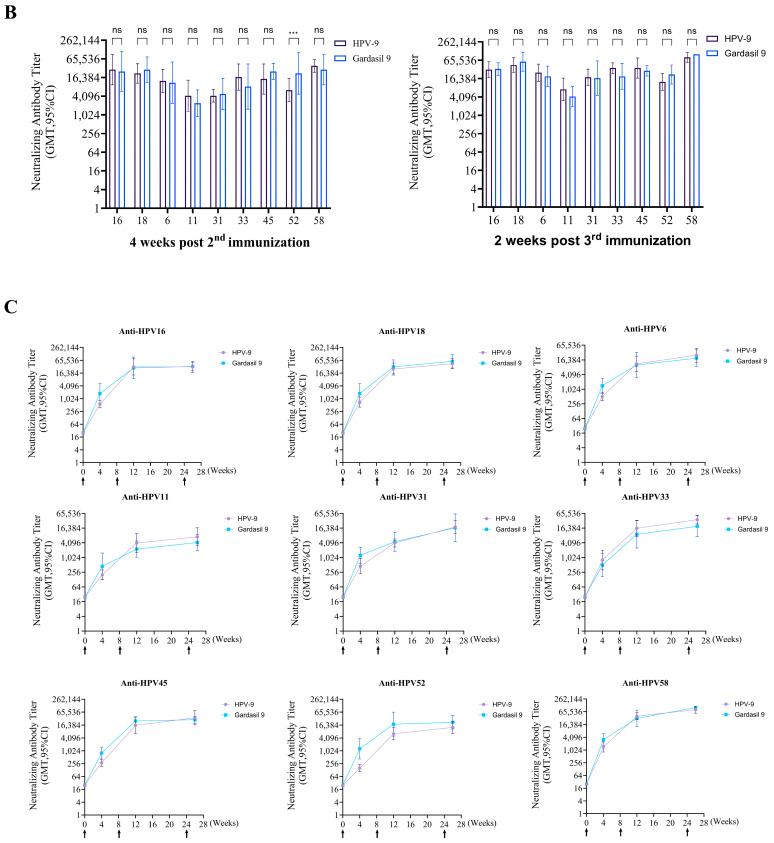
Comparing the immunogenicity of the HPV-9 vaccine with that of Gardasil^®^ 9 in macaques. (**A**) Macaques were randomly divided into groups (3 females and 3 males in each group), as the immunization routine scheme shows. Serum samples were collected as the control, followed by intramuscular injection of 1 clinical dose of the HPV-9 vaccine or Gardasil at weeks 0, 8, and 24 and the collection of blood at weeks 4, 12, and 26. (**B**) After the 2nd or 3rd vaccination, neutralizing antibody titers were detected at weeks 12 and 26, which indicated that the immunogenicity of the HPV-9 vaccine is comparative to that of Gardasil^®^ 9 (*p* > 0.05 (ns), and ≤0.001 (***)). (**C**) Trend regarding neutralizing antibodies at each timepoint compared with Gardasil^®^ 9. The arrowheads represent the vaccination timepoints.

In order to investigate and compare the immunogenicity of the HPV-9 vaccine with that of Gardasil^®^ 9 in a non-human primate (NHP) model, macaques were randomly grouped (*n* = 6) and intramuscularly injected with one clinical dose of either vaccine at weeks 0, 8, and 24. Serum samples were collected at weeks 4, 12, and 26 to measure neutralizing antibody titers, with pre-vaccination sera serving as the negative control (Figure 5A). By week 12 (after two vaccinations), the HPV-9 vaccine effectively induced neutralizing antibodies against HPV 6, 11, 16, 18, 31, 33, 45, 52, and 58, with values of 11,404; 4032; 32,254; 25,600; 4032; 16,127; 16,127; 6400; and 40,637, respectively. Gardasil^®^ 9 induced comparable values of 10,159; 2263; 28,735; 32,254; 4525; 905; 25,600; 18,102; and 32,254 (Figure 5B). Upon three-time vaccination, the HPV-9 vaccine induced comparable antibody levels to Gardasil^®^ 9, inducing antibody values of 25,600; 7184; 32,254; 45,614; 7184; 18,102; 36,204; 36,204; 12,800; and 81,275 against HPV types 6, 11, 16, 18, 31, 33, 45, 52, and 58, respectively. In the Gardasil^®^ 9 group, the corresponding antibody values were 19,401; 4222; 33,779; 58,813; 16,889; 19,401; 29,407; 22,286; and 102,400 (Figure 5B). The administration of the HPV-9 significantly induced antigen-specific neutralizing antibodies starting from 4 weeks after the first vaccination. Despite significantly higher titers against HPV types 16, 6, 31, 45, and 52 being induced by Gardasil^®^ 9 at week 4, the HPV-9 vaccine could induce comparable neutralizing antibody titers against all nine HPV types at weeks 12 and 26, with no significant differences being observed (Figure 5C). These results demonstrate that the HPV-9 vaccine can induce immunogenicity comparable to Gardasil^®^ 9 in macaques, aligning with the findings derived from the use of rodent models.

## 4. Discussion

Currently, polyvalent HPV vaccines targeting multiple HPV types have been approved for clinical use, with several more candidates undergoing clinical trials or preclinical studies. Among these vaccines, our HPV-9 vaccine was developed based on the bivalent vaccine Walrinvax^®^, which has been approved by the National Medical Products Administration (NMPA) in China, and the WHO prequalification was received in 2024. Compared with the bivalent HPV vaccine, nine-valent HPV vaccines can provide a wider range of protection. In addition to preventing cervical cancer, the HPV vaccine is of great significance in the prevention and control of a variety of HPV-related diseases, including genital warts, anal cancer, vaginal cancer, and oropharyngeal cancer [18,19]. Most cases of cervical, anal, and oropharyngeal cancer are caused by HPV infection and the development of the HPV vaccine has led to a dramatic reduction in cases of HPV-related illness [20]. In addition, further development of HPV-based therapeutic vaccines is necessary. For patients who have been infected with HPV, therapeutic vaccines can help remove the virus from the body and inhibit tumor growth [21,22].

To develop the HPV-9 vaccines, recombinant VLPs were utilized as antigens. Prior to the adsorption of the AlPO_4_ adjuvant, comprehensive structural characterization of the VLPs was conducted. Immunogenicity studies of the monovalent VLPs in mice revealed that even at a low dose of 0.02 µg, the recombinant VLPs were capable of provoking excellent humoral responses. Both binding antibodies and neutralizing antibodies against the nine targeted HPV types exhibited a dose-dependent relationship (Figure 2B,C). Overall, these data provide support for the development of the HPV-9 vaccine utilizing recombinant VLPs as the antigenic components.

The immunogenicity of the HPV-9 vaccine was assessed in multiple animal models, including both rodent- and non-human primate-based models. Vaccination with the HPV-9 vaccine three times effectively induced antigen-specific neutralizing antibodies in both BALB/c mice (Figure 3B) and SD rats (Figure 4B). Notably, the neutralizing antibody titers reached their maximum levels two weeks after the third vaccination, at week 6. Although there was a subsequent decline in titers, they remained at elevated levels for a prolonged duration (Figure 3C and Figure 4C). Importantly, when comparing the immunogenicity of the HPV-9 with that of Gardasil^®^ 9 in macaques, the HPV-9 vaccine could elicit a comparable immune response. Although vaccination with the HPV-9 vaccine twice induced significantly lower neutralizing antibody titers against the pseudovirus of HPV52 at 4 weeks after the second immunization, the HPV52-specific neutralizing antibody titers were comparable to those induced by Gardasil^®^ 9 at 2 weeks following the third immunization. These results strongly suggest that the HPV-9 vaccine could provide high immune efficacy and excellent durability of neutralizing antibodies against infection of the nine HPV types, having effects comparable to Gardasil^®^ 9 and Walrinvax^®^.

The ELISA performed to detect anti-HPV antibodies (the results of which are shown in Figure 2) is based on HPV VLPs produced and expressed in the *Pichia pastoris* expression system. The difference in the response of Gardasil^®^ 9 to coated VLPs may arise from the use of a different expression system or a different L1 protein sequence. For these reasons, antibodies induced by HPV-9 vaccine may be more specific for coated VLPs than those of Gardasil^®^ 9. Furthermore, neutralizing antibodies are active ingredients that protect against HPV infection and are the gold standard for evaluating the effectiveness of HPV vaccines. Therefore, we opted for a pseudovirus-based neutralization experiment to compare the immunogenicity of the HPV-9 vaccine and Gardasil^®^ 9.

The neutralizing antibodies induced by the HPV-9 vaccine against the pseudovirus of HPV types 33 and 45 are significantly higher than those of Gardasil^®^ 9, owing to the fact that the L1 sequences of HPV 33 and HPV 45 pseudovirus used in this study were homologous to the L1 proteins contained in the HPV-9 vaccine. For HPV 45, there were 4 amino acids different from the relevant patent of Gardasil^®^ 9, of which the residue at site 357 in the HI Loop was critical for neutralizing antibody sensitivities [23]. Currently, the antigen sequence of type 33 in Gardasil^®^ 9 has not been identified. The L1 sequence of HPV 33 that we used was different from that of the HPV33 pseudovirus constructed by Richard Roden, which contains 6 mutations; the differences at sites 133 (DE loop) and 266 (FG loop) were critical for neutralizing antibody sensitivities [24,25]. Except for types 33 and 45, other types of pseudoviruses share the same L1 sequence as Gardasil 9, so we believe these results are relatively accurate. Overall, the HPV-9 vaccine may provide effective protection against HPV infection.

Although HPV-9 comprises the same types as Gardasil^®^ 9 with the same dose of antigens, the L1s used in our HPV-9 vaccine are expressed in *Pichia pastoris,* while the L1s of Gardasil^®^ 9 are expressed in *Saccharomyces cerevisiae.* In the *Pichia pastoris* expression system, the length of oligosaccharide chains added through post-translational glycosylation is very similar to the structure of higher eukaryotes, resulting in strong immunogenicity. Compared to *Saccharomyces cerevisiae*, the *Pichia pastoris* expression system allows for higher expression levels and high-density cell culture. The *Saccharomyces cerevisiae* expression system is prone to plasmid loss and unstable passages, while all the expression vectors of the *Pichia pastoris* expression system must be integrated into the yeast genome. Therefore, the *Pichia pastoris system* has many advantages, such as simplified production conditions, lower production costs, and potent immunogenicity, making it valuable for various applications with significant market potential.

In summary, we have successfully developed a nine-valent vaccine candidate, the HPV-9 vaccine, which is capable of eliciting an immune response comparable to that of Gardasil^®^ 9. This vaccine candidate holds promise in providing protection against infections caused by the nine types of HPV and is currently being tested in a phase III clinical trial (NCT05580341). This large-scale phase III clinical trial will further demonstrate the safety and efficacy of the HPV-9 vaccine. However, the HPV-9 vaccine and the currently marketed HPV vaccine are prophylactic vaccines, which are mainly used to prevent infection before exposure to the virus and cannot be used to clear existing HPV infection. Unfortunately for patients who are already infected with HPV, such vaccines are not effective against existing infections and cannot reverse the lesions caused by the infection. Moreover, vaccines can only prevent HPV infection and the cancers it can cause; they do not provide a therapeutic effect for patients who have developed precancerous lesions or cancer. Therefore, in follow-up studies, we will continue to develop HPV-based therapeutic vaccines to help the immune system of infected patients clear HPV infection and reverse the lesions caused by HPVs. Therapeutic vaccines can also be combined with other immunotherapies to improve treatment effectiveness.

## 5. Conclusions

In this study, we developed a nine-valent recombinant HPV virus-like particle (VLP) vaccine (the HPV-9 vaccine). Our findings demonstrate that the HPV-9 vaccine elicits a robust and long-lasting neutralizing antibody response, effectively preventing HPV infection caused by HPV types 6, 11, 16, 18, 31, 33, 45, 52, and 58. Moreover, the HPV-9 vaccine is comparable to Gardasil^®^ 9, which is already available on the market. As a potential second-generation prophylactic HPV vaccine, this novel formulation holds promise in terms of reducing the financial burden associated with global vaccine supply. Of course, we will aim to further prove the safety and efficacy of this vaccine in phase III clinical trials.

## Data Availability

The data presented in this study are available on request from the corresponding author. This study did not generate any unique code.
